# Immunological and pathological roles of Siglecs: a molecular review

**DOI:** 10.3389/fimmu.2026.1826359

**Published:** 2026-05-21

**Authors:** Lakshmi K, Vino Sundararajan

**Affiliations:** Integrative Multiomics Lab, Department of Bio Sciences, School of Bio Sciences and Technology, Vellore Institute of Technology, Vellore, Tamil Nadu, India

**Keywords:** autoimmune, cancer immune evasion, glycoimmune checkpoints, inflammatory diseases, neurodegeneration, Siglec ITIM/ITAM signaling

## Abstract

Siglecs are sialic acid-binding immunoglobulin-like receptors that regulate immune responses through inhibitory and activating signaling pathways. By recognizing sialoglycan ligands, they modulate innate and adaptive immunity, and their dysregulation is increasingly linked to diverse human diseases. This review highlights the roles of human Siglecs in disease pathogenesis, including cancer, autoimmune and inflammatory disorders, neurodegeneration, and infections. We summarize key regulatory mechanisms such as transcription factors, microRNAs, and protein interactions and outline major signaling pathways underlying Siglec-mediated immune modulation. At the molecular level, Siglecs signal via ITIM dependent inhibitory pathways SHP-1/SHP-2 or ITAM/DAP12-associated activation SYK/MAPK, maintaining immune homeostasis. Targeting the Siglec glycan axis offers promising therapeutic opportunities.

## 1 Introduction

**Figure 1:**
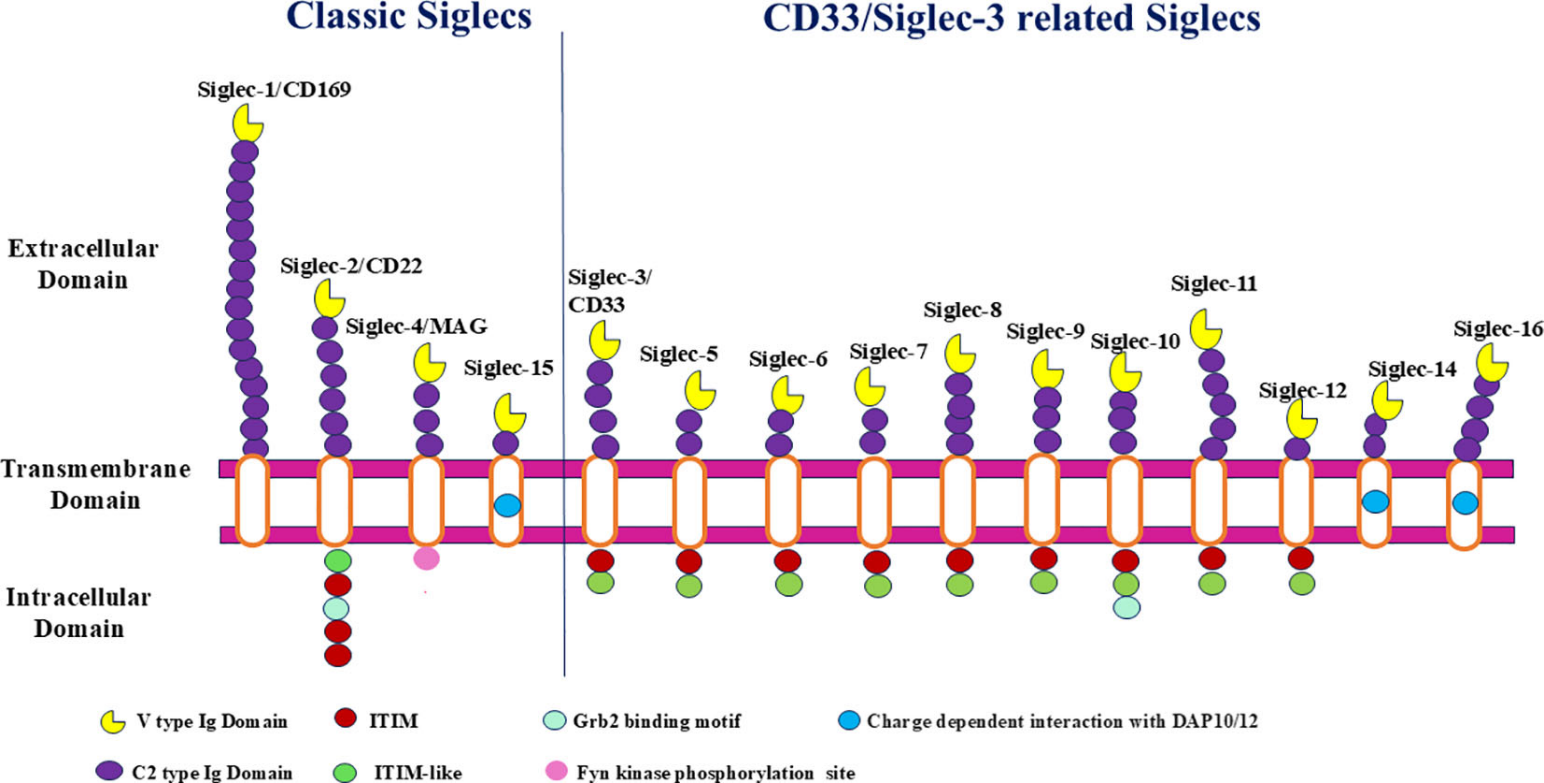
Structural domains of classic and CD33/Siglec-3 related Siglecs, highlighting extracellular Ig domains with intracellular signaling cascades.

Human Siglecs, also known as sialic acid-binding immunoglobulin-like lectins, are immune-regulatory receptors that mediate glycan recognition and signal transduction (1). They primarily recognize sialic acids, particularly N-acetylneuraminic acid (Neu5Ac), an acidic nine-carbon monosaccharide commonly found at the terminal positions of glycoproteins and glycolipids (2). Through this recognition, Siglecs regulate cell-cell communication, immune signaling, and host pathogen interactions, thereby influencing immune homeostasis and disease susceptibility (3). Siglecs are encoded within a distinct gene cluster and share a conserved structural organization. Structurally, Siglecs comprise an extracellular region containing immunoglobulin-like domains, a single-pass transmembrane domain, and a cytoplasmic tail involved in signal transduction **Figure 1**. The extracellular region includes an N-terminal V-set domain responsible for sialic acid binding, largely mediated by a conserved arginine residue, followed by one or more C2-set domains that contribute to receptor stability rather than ligand binding (4).

Functionally, Siglecs can transmit either inhibitory or activating signals depending on their intracellular and transmembrane features. Most CD33-related Siglecs (e.g., Siglec-7, -9, and -10) contain immunoreceptor tyrosine-based inhibitory motifs (ITIMs) or ITIM-like domains within their cytoplasmic tails. Upon engagement with sialoglycans, these motifs recruit phosphatases such as SHP-1 and SHP-2, resulting in attenuation of activating signaling pathways and suppression of immune cell activation (4, 5). In contrast, a subset of Siglecs contains positively charged residues within their transmembrane domains that enable association with the adaptor protein DNAX-activating protein of 12 kDa (DAP12), which carries immunoreceptor tyrosine-based activating motifs (ITAMs). Phosphorylation of ITAMs results in SYK kinase recruitment and activation of downstream signaling pathways, including MAPK, thereby amplifying inflammatory and innate immune responses (4, 5). Notably, several Siglecs exist as paired receptors with highly similar extracellular domains but divergent intracellular signaling motifs, allowing them to deliver opposing inhibitory or activating signals. Maintenance of this balance is essential for fine-tuned immune regulation (6). In addition, Siglec gene expression and function are modulated by protein interaction partners, microRNAs, and transcription factors, linking Siglec signaling to broader regulatory networks involved in disease pathogenesis.

Despite increasing interest, the precise contributions of Siglecs to human diseases including cancer, autoimmune disorders, neurodegenerative conditions, and infectious diseases remain incompletely understood. Limited knowledge of Siglec ligand interactions and disease-specific immune functions continues to hinder their therapeutic exploitation. This review therefore aims to consolidate current understanding of human Siglec biology in disease contexts and to highlight emerging molecular mechanisms and future research directions (7).

## 2 Human Siglec members

Human Siglecs are immune regulatory receptors that recognize sialic acids and modulate immune signaling. Based on the specific structural features of their transmembrane and cytoplasmic domains, human Siglecs are broadly classified into inhibitory and activating receptors.

Human Siglecs include conserved members Siglec-1/CD169, Siglec-2 (CD22), Siglec-4 (MAG), and Siglec-15 and the CD33 related Siglecs (Siglec-3, -5, -6, -7, -8, -9, -10, -11, -12, and -16). Siglec-1/CD169 and Siglec-4 lack classical cytoplasmic signaling motifs, whereas most CD33-related Siglecs possess immunoreceptor tyrosine-based inhibitory motifs (ITIMs). In contrast, Siglec-14, Siglec-15, and Siglec-16 are activating Siglecs that associate with the adaptor protein DAP12 through positively charged transmembrane residues. Siglec-13 is absent in humans (3, 8).

### 2.1 Inhibitory Siglecs

Inhibitory Siglecs maintain immune homeostasis by limiting excessive immune activation through interactions with sialoglycan ligands. This interaction helps guard against overactivation of the immune system (1). Inhibitory Siglecs possess one or more Tyrosine residues located inside the cytoplasmic tails of ITIM motifs. When a sialylated ligand attaches to an inhibitory Siglec, the ITIMs undergo phosphorylation and attract downstream effector protein(s). Inhibitory Siglecs typically recruit phosphatases (e.g., SHP-1/2) to suppress immune activation, not stimulate it (4). Several inhibitory Siglecs are implicated in disease pathogenesis. Siglec-2 regulates B-cell differentiation and is linked to lymphoma (9). Pathogens such as *Neisseria gonorrhoeae* exploit Siglec-9 to evade innate immunity, and elevated Siglec-9 expression is associated with rheumatoid arthritis (10). In neuroinflammatory disorders, Siglec-1/CD169 mediated signaling in microglia contributes to disease progression (11). Additionally, Siglec-11 reduces microglial neurotoxicity through interactions with neuronal gangliosides (12).

### 2.2 Activator Siglecs

Siglecs play a crucial role in the progression of several diseases. In contrast, activating Siglecs associate with the adaptor protein DAP12 through positively charged residues within their transmembrane domains. Ligand binding induces phosphorylation of DAP12 ITAM motifs, resulting in recruitment and activation of spleen tyrosine kinase (SYK) and subsequent modulation of downstream signaling pathways in a cell type dependent manner (13). Siglec-14 exhibits context-dependent immune functions, and deletion of the Siglec-14 gene has been linked to protection against inflammatory diseases such as tuberculosis meningitis and COPD exacerbations. Its functional pairing with inhibitory Siglec-5 highlights a balance between immune activation and suppression (14).

**Figure 2:**
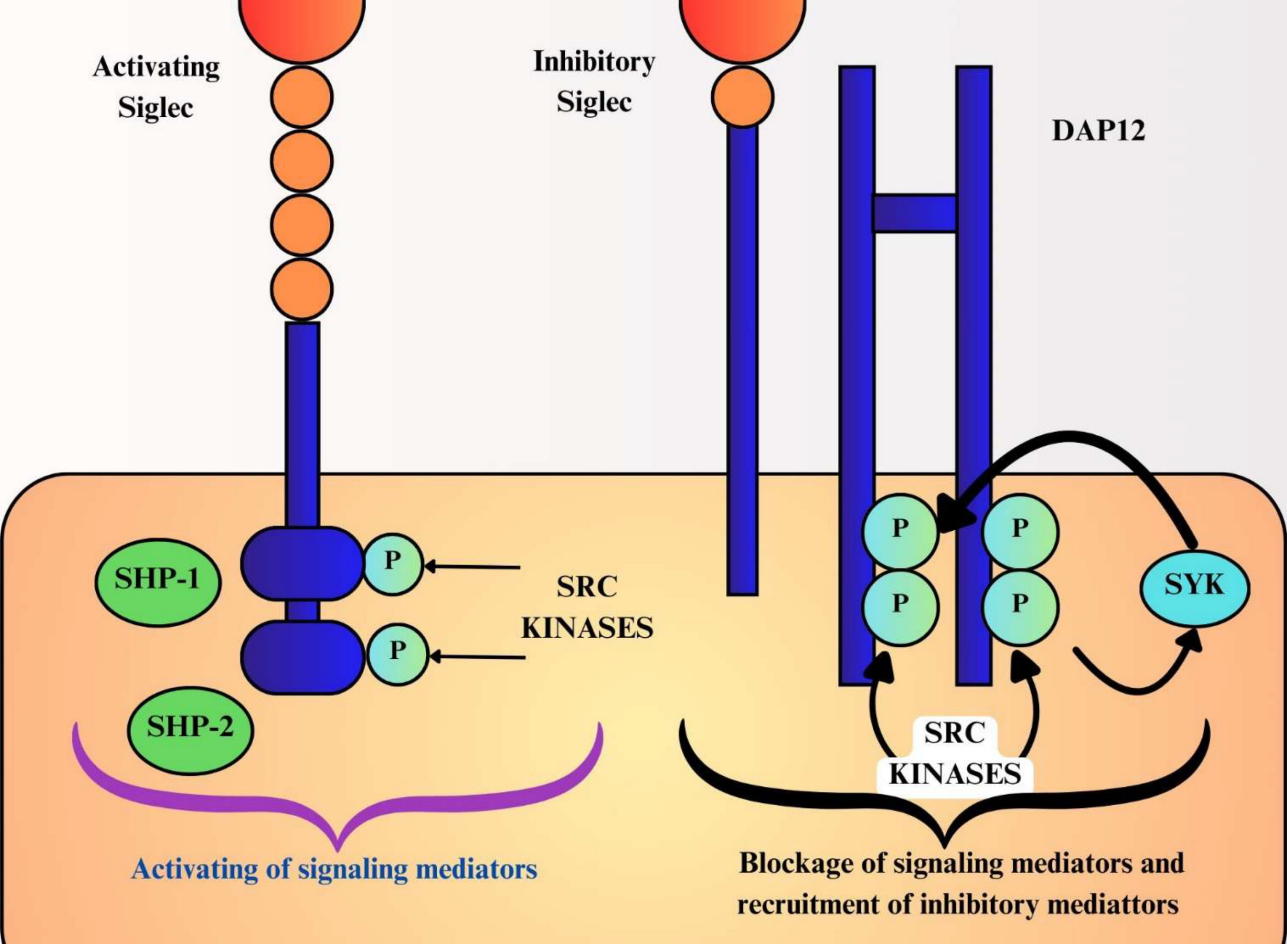
The mode of action of activating and inhibitory Siglecs in mitigating the progression of diseases.

Siglec-15 acts as a glyco-immune checkpoint molecule and is upregulated in several cancers, including leukemia, lung adenocarcinoma, and glioma. By promoting an immunosuppressive tumor microenvironment, Siglec-15 represents a promising therapeutic target, particularly in tumors resistant to PD-1/PD-L1 blockade (15, 16). Human Siglec-16 is an ITAM-associated receptor that forms a paired system with Siglec-11. Despite their high sequence similarity in the extracellular domain and shared recognition of the ligand polysialic acid (polySia), the two receptors mediate opposing signaling outcomes (17). In the context of neuroinflammatory regulation, Siglec-11 functions as an inhibitory receptor, dampening inflammatory responses through ITIM-dependent signaling pathways. In contrast, Siglec-16 promotes proinflammatory signaling via DAP12-associated ITAM pathways upon engagement with polySia (6). **Figure 2** highlights these contrasting mechanisms of inhibitory and activating Siglecs and their roles in disease regulation.

## 3 Siglec-mediated immune signaling: beyond simplistic inhibitory models

Siglecs function as key modulators of immune homeostasis through their ability to recognize sialylated glycans expressed on host and pathogen surfaces. Rather than acting as classical ligand-activated receptors, Siglec signaling is highly context-dependent and is influenced by receptor clustering, ligand density, and the cellular microenvironment (18). Most CD33-related Siglecs (e.g., Siglec-7, -9, and -10) contain immunoreceptor tyrosine-based inhibitory motifs (ITIMs) or ITIM-like domains within their cytoplasmic tails. Upon engagement with sialoglycans, these motifs recruit phosphatases such as SHP-1 and SHP-2, resulting in attenuation of activating signaling pathways and suppression of immune cell activation. Importantly, emerging evidence demonstrates that the sialic acid-Siglec axis functions as a glyco-immune checkpoint, analogous yet distinct from classical checkpoints such as PD-1/PD-L1. This pathway plays a central role in tumor immune evasion, where hypersialylation of cancer cells enhances Siglec engagement on myeloid and lymphoid cells, leading to broad immunosuppressive effects within the tumor microenvironment (19, 20).

Notably, not all Siglecs conform to inhibitory paradigms. For example, Siglec-15 lacks canonical ITIM domains and instead signals through adaptor molecules such as DAP12, which contains immunoreceptor tyrosine-based activation motifs (ITAMs). Despite this, Siglec-15 paradoxically exerts immunosuppressive functions, particularly by inhibiting T-cell proliferation and modulating macrophage activity. This highlights the complexity of Siglec signaling and suggests that functional outcomes depend on receptor context rather than motif classification alone (15, 21). Recent studies further indicate that Siglec engagement regulates multiple downstream pathways, including cytokine production, antigen presentation, and chemokine signaling (e.g., CCL2-mediated T-cell suppression), reinforcing its role as a central regulator of immune tolerance and inflammation (22). Collectively, these findings underscore that Siglec signaling is not a linear pathway but a multi-layered regulatory network integrating glycan recognition with immune checkpoint control.

## 4 Siglec gene role in human diseases

Multiple Siglecs recognize sialic acids through a conserved interaction between the sialic acid carboxyl group and an arginine residue within the N-terminal Ig V-set domain. Siglec function is influenced by both *cis* and *trans* ligand interactions, with *cis* interactions generally exerting a stronger regulatory effect without completely blocking *trans* engagement. Through recognition of sialylated glycans, which act as self-antigen, Siglecs enable immune cells to distinguish self from non-self and modulate cellular activation states (3, 23, 24).

Siglecs are expressed on multiple immune cell types, including B cells, T lymphocytes, dendritic cells, and macrophages, where they regulate both innate and adaptive immune responses (13) In B cells, inhibitory Siglecs such as Siglec-2 and Siglec-10 function as co-receptors of the B-cell receptor, promoting B-cell tolerance by suppressing or eliminating autoreactive clones. Similarly, Siglecs modulate dendritic cell activation and cytokine production, thereby shaping immune outcomes (14). Several pathogens exploit Siglec sialic acid interactions to evade host immunity by decorating their surfaces with host-like sialylated glycans, engaging inhibitory Siglecs and dampening immune responses. In contrast, certain Siglecs, including Siglec-1/CD169 and Siglec-14, can recognize pathogen-associated sialic acids and enhance host defense mechanisms (24, 25). Siglec-1/CD169, in particular, influences macrophage activation and can induce either pro-inflammatory or tolerogenic responses depending on the ligand context. Although most human T cells do not express Siglecs, subsets of suppressor T cells release soluble CD52, which can bind Siglec-10 and suppress T-cell activation, contributing to immune regulation in autoimmune conditions. However, the precise molecular mechanisms underlying this interaction remain to be fully elucidated (26). Overall, by fine-tuning immune activation and tolerance, Siglecs play critical roles in infection, autoimmunity, and immune homeostasis, highlighting their potential as therapeutic targets in human diseases.

### 4.1 Siglec genes in autoimmune diseases

Siglecs play important immunoregulatory roles in autoimmune diseases by modulating immune cell activation and tolerance, and several members serve as biomarkers of disease activity. Among them, Siglec-1/CD169 is a type I interferon inducible adhesion molecule expressed on monocytes and tissue macrophages and forms part of the interferon-regulated gene signature. Elevated Siglec-1/CD169 expression correlates strongly with disease activity in rheumatoid arthritis (RA) and systemic lupus erythematosus (SLE), and it has proven clinical utility as a biomarker in juvenile dermatomyositis and systemic sclerosis (27). Siglec-1/CD169 has also been detected in the urine of SLE patients and in circulating monocytes as a marker of type I interferon activity in juvenile dermatomyositis (28). Siglec-1/CD169 expression is associated with interferon-related transcription factors such as IRF9 and STAT1 in primary Sjögren’s syndrome, and increased levels have been observed in monocytes from individuals with type 1 diabetes, suggesting its potential value in early diagnosis and therapeutic monitoring (29, 30). Additionally, abundant Siglec-1/CD169 myeloid cells are present in active multiple sclerosis lesions, implicating Siglec-1/CD169 in autoimmune and neuroinflammatory processes of the central nervous system (11).

Siglec-2 (CD22) also contributes to immune homeostasis in autoimmunity. In experimental autoimmune encephalomyelitis, Siglec-2 signaling exerts a protective role by regulating microglial activation, whereas its inhibition exacerbates disease severity and promotes proinflammatory M1 polarization (31). Clinically, soluble Siglec-2 levels correlate with disease severity and B-cell frequency in myasthenia gravis, while reduced plasma soluble CD22 is associated with poorer outcomes in IgA nephropathy (32, 33). Genetic variation in Siglec-3 (CD33), including a 19-base pair deletion, has been identified as a potential risk factor for neuromyelitis optica spectrum disorders. Increased Siglec-6 expression has also been reported in lupus nephritis, particularly in patients experiencing recurrent disease flares (34, 35).

Emerging evidence further highlights the CD24 Siglec axis as a key immunoregulatory checkpoint in autoimmune and inflammatory conditions (33). CD24 is a highly sialylated glycoprotein expressed on immune and epithelial cells. It has been reported to interact with Siglec-3 and, possibly, Siglec-10 on myeloid and regulatory immune cells to suppress excessive inflammatory responses. This pathway contributes to immune tolerance and protection against autoimmune pathology and inflammatory complications, including severe respiratory inflammation. (36). Early clinical studies of CD24Fc (NCT02650895) suggest that the CD24 Siglec pathway regulates lipid metabolism and inflammation in humans, highlighting its potential as a therapeutic target for metabolic disorders (37). CD24, a GPI-anchored glycoprotein, mediates inflammatory responses through interactions with Siglecs (33). Collectively, these findings underscore the diverse and context dependent roles of Siglecs in autoimmune diseases, supporting their potential as biomarkers and therapeutic targets.

### 4.2 Siglec members in cancer

Tumor cells undergo extensive glycosylation changes, collectively termed the tumor glycome or glycocode, characterized by increased expression of sialic acids such as N-acetylneuraminic acid (Neu5Ac), N-glycolylneuraminic acid (Neu5Gc), and tumor-associated sialylated antigens including sialyl-Tn (sTn). These aberrant glycans act as ligands for Siglecs on immune cells, facilitating immune evasion within the tumor microenvironment. Notably, sTn binding to Siglec-15 on myeloid cells activates DAP12-SYK signaling and increases TGF-β secretion, promoting an immunosuppressive microenvironment (38). Siglecs contribute to cancer progression and immune regulation by functioning as biomarkers, immune checkpoints, and therapeutic targets. Siglec-1/CD169 expression on lymph node macrophages supports the activation of antitumor cytotoxic T lymphocyte responses (39). In B-cell precursor acute lymphoblastic leukemia, targeting Siglec-2 with the antibody drug conjugate inotuzumab ozogamicin selectively induces DNA damage and tumor cell death (40). CD22-targeting bispecific antibodies (TCBs) show that antibody avidity and binding domain determine cytotoxicity, with optimized TCBs like G5/44 effectively engaging T cells and suppressing tumor growth (41). A CAR T platform secreting a CD33-directed bispecific engager (7033) overcomes AML antigen escape and heterogeneity, enhancing T-cell activation, persistence, and antitumor efficacy (42). Likewise, allogeneic CD33-directed CAR-NKT cells exhibit robust bone marrow homing, effectively target BM-resident and CD33-low leukemia cells, synergize with hypomethylating agents, and show minimal off-tumor toxicity and reduced risk of graft-versus-host disease, underscoring their therapeutic potential in myeloid malignancies (43). Siglec-6 expressing AXL^+^ dendritic cells have been identified in inflammatory tumor tissues, including colorectal cancer, and Siglec-6/CD3 bispecific antibodies show promise in eliminating chronic lymphocytic leukemia cells in preclinical models (44, 45). Interaction of Siglec-7 on natural killer cells with *Fusobacterium nucleatum* RadD suppresses NK cell-mediated cytotoxicity, revealing a novel microbial-driven immune evasion mechanism (46). Engineering Siglec-9 based chimeric switch receptors enhances cytokine release and antitumor activity, highlighting their therapeutic potential, while Siglec-9 expression also correlates with prognosis in cutaneous melanoma (47, 48).

**Figure 3:**
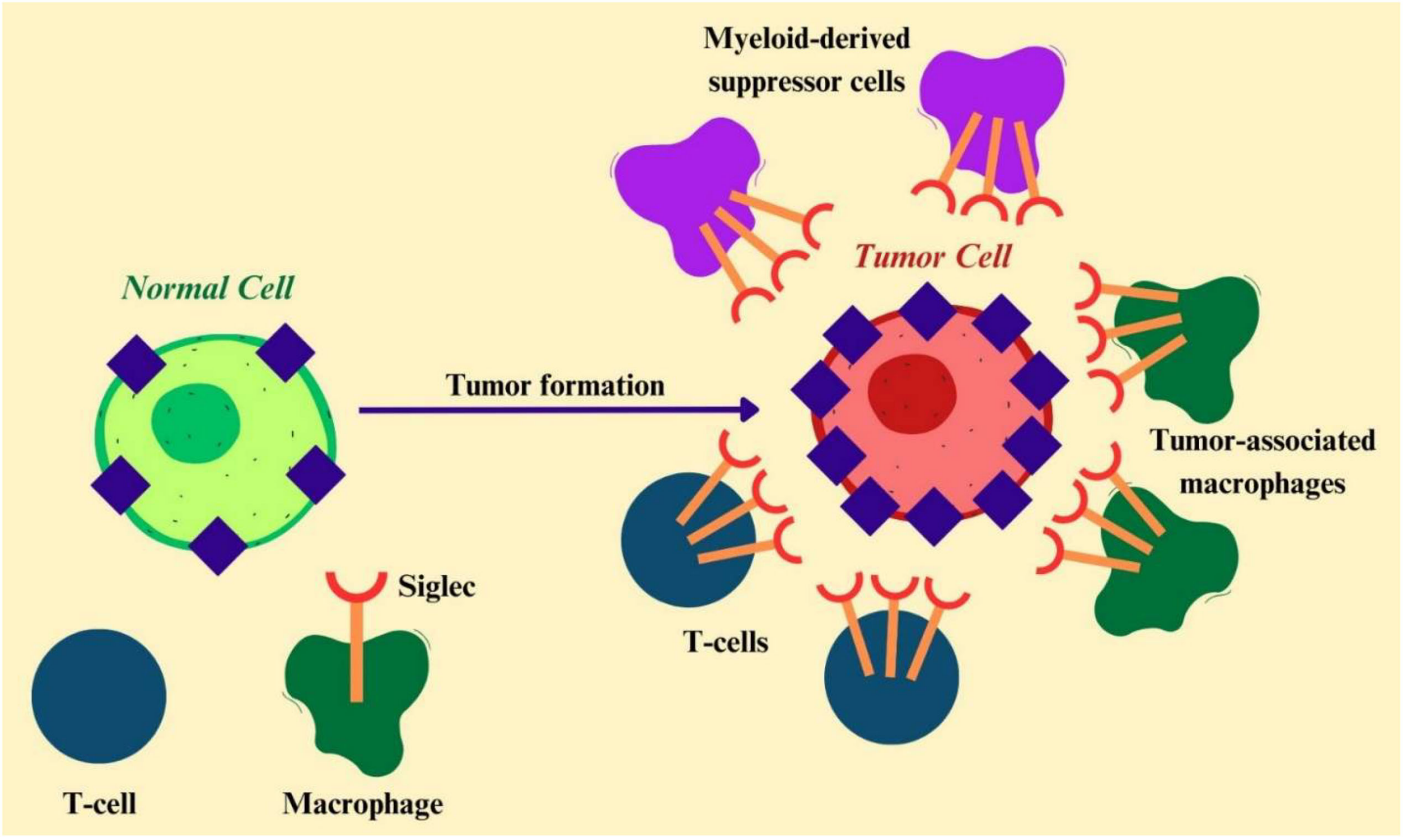
Modified expression of Siglecs on immune cells and their interactions with tumor cells.

Siglec-10 binds sialylated glycans via its D1 domain and forms D2-mediated homodimers that enhance tumor cell interactions; however, recent studies indicate it does not specifically bind CD24, suggesting broader ligand recognition (49). Consistently, CRISPRa screening shows that Siglec-10 ligand expression is regulated by glycosylation pathways and is independent of CD24, with GAL3ST4 identified as a potential immune evasion driver (50). In PDAC, Siglec-10 engagement with alternative sialylated ligands such as integrin α3β1 suppresses macrophage phagocytosis and promotes tumor progression, while its blockade restores antitumor immunity (51). Although earlier studies proposed CD24 as a key Siglec-10 ligand mediating a “don’t eat me” signal and a therapeutic target (52, 53), emerging evidence suggests that this interaction may be context-dependent or dispensable. Siglec-12 expression is associated with oncogenic features in a subset of mismatch repair deficient colorectal cancers (54). Importantly, Siglec-15 has emerged as a glyco-immune checkpoint molecule. Its expression, partly regulated by the aryl hydrocarbon receptor, limits T-cell infiltration and promotes immune evasion in several cancers, including head and neck squamous cell carcinoma, and it has been proposed as a prognostic biomarker in lung adenocarcinoma (55, 56). Multifaceted roles of Siglecs in shaping tumor immunity, cancer progression, and therapeutic responsiveness. **Figure 3** schematically illustrates altered Siglec expression on immune cells and their interactions with tumor cells, highlighting the downstream consequences for immune regulation and tumor advancement.

### 4.3 Neurodegenerative disease

Siglecs play critical immunoregulatory roles in the central nervous system (CNS), where they are predominantly expressed on microglia. Dysregulation of the sialic acid Siglec axis, particularly impaired sialylation, has been associated with chronic neurodegenerative disorders such as Alzheimer’s disease (AD). Soluble low molecular weight polySia (avDP20) interacts with SIGLEC-11 and exerts anti-inflammatory effects on human THP-1 macrophages (57). Reduced plasma levels of soluble Siglec-2 correlate with decreased cerebrospinal fluid Aβ42 and cognitive impairment, suggesting its association with AD pathology. Siglec-2 also interacts with the insulin-like growth factor 2 receptor (IGF2R), influencing lysosomal trafficking and implicating potential relevance in Niemann-Pick type C disease (58, 59). CD33 regulates microglial activity, and it’s signaling likely involves ligand-induced receptor clustering rather than a simple monomer-dimer switch (60). Siglec-3 (CD33) has been strongly linked to AD through its role in microglial regulation and amyloid plaque compaction. Elevated peripheral expression of Siglec-3 correlates with inflammatory biomarkers and disease progression, supporting a causal role in AD pathogenesis. Additional Siglecs, including Siglec-6, Siglec-8, Siglec-9, Siglec-10, Siglec-11, and Siglec-14, have also been implicated in microglial activation, neuroinflammation, and CNS dysfunction, further underscoring the importance of Siglecs in neurodegenerative disease mechanisms (61). Collectively, Siglecs are essential for maintaining immune homeostasis in the CNS, and their dysregulation contributes to neurodegenerative disease progression.

### 4.4 Infectious diseases

Siglecs play diverse roles in host-pathogen interactions by modulating innate and adaptive immune responses during infection. Siglec-1/CD169 is particularly important in myeloid cells, where it links innate and adaptive immunity through antigen capture and presentation in viral infections such as respiratory syncytial virus and HIV (62). Nanoclustering of Siglec-1/CD169 enhances binding to sialylated viral particles, promoting HIV-1 capture and transmission.

Siglec-2 and Siglec-3 influence antiviral immunity in chronic hepatitis B and HIV. Modulation of Siglec-2 enhances cytokine production and antigen presentation suppressed during viral infection. Siglec-3 limits HIV-1 replication in macrophages and T cells and represents a potential therapeutic target for viral eradication strategies (63). Siglec-6, expressed on AXL^+^ dendritic cells, contributes to T-cell polarization and viral transmission in HIV infection (44). Siglec-7 and Siglec-9 regulate interactions with viral pathogens and modulate natural killer cell cytotoxicity, affecting disease outcomes in hepatitis C and HIV (44). Siglecs are also exploited by bacterial pathogens. *Neisseria gonorrhoeae* uses sialylated lipooligosaccharides to engage inhibitory Siglecs on neutrophils, suppressing antimicrobial responses and complement activation, thereby facilitating immune evasion (64). Together, these findings highlight Siglecs as key regulators of host immunity and promising targets for therapeutic intervention in infectious diseases.

### 4.5 Covid-19

Siglecs play important roles in regulating immune responses during SARS-CoV-2 infection. Elevated Siglec-1/CD169 expression on monocytes has been observed in patients with moderate COVID-19 compared to severe cases, suggesting a protective immunoregulatory role. Transcriptomic analyses of SARS-CoV-2-positive samples reveal significant upregulation of macrophage-associated genes, including Siglec-1/CD169, indicating its involvement in antiviral immunity (65). Siglec-1/CD169 mediates ACE2-independent SARS-CoV-2 entry into macrophages, triggering pro-inflammatory cytokines (TNFα, IL-6, IL-1β) without productive infection, which is suppressed by remdesivir; blocking Siglec-1/CD169 may reduce macrophage-driven hyperinflammation in COVID-19 (66). Siglec-3 serves as a biomarker for stratifying COVID-19 severity, distinguishing moderate from severe disease (67). Siglec-7 and its counterreceptor CD45 have also been associated with disease severity, highlighting the role of inhibitory Siglec pathways in immune dysregulation during infection (68). Therapeutic targeting of Siglec-8 has been proposed to reduce eosinophil and mast cell driven hyperinflammation in COVID-19 (69).

Siglec-9 exhibits dual functions in COVID-19. While it suppresses NK cell cytotoxicity, blockade of Siglec-9 enhances antibody-dependent cellular cytotoxicity against SARS-CoV-2 infected cells, improving immune clearance (70). Conversely, Siglec-9 agonism may mitigate neutrophil-driven hyperinflammation. Notably, interaction between Siglec-9 and the SARS-CoV-2 Omicron receptor-binding domain impairs macrophage phagocytosis and antigen presentation, an effect partially reversed by viral mutations such as F375S (71, 72). Overall, Siglecs emerge as critical modulators of immune balance in COVID-19 and represent promising targets for therapeutic intervention. Additional roles of Siglecs in human diseases have been summarized in **Table 1**.

**Table 1:** Role of inhibitory and activator Siglecs in human health and diseases.

Siglec genes	Disease	Role	Reference
Siglec-1/CD169	Rheumatoid arthritis	Elevated in macrophages and monocytes. Linked to the transcriptional pattern of IFNs. Shows a connection with the clinical activity of rheumatoid arthritis. Candidate gene regulated by type I interferons (26).	(73, 74)
	Systemic Lupus Erythematosus	Monocytes and macrophages regulated by sialic acids on cell surfaces, and its association with IFNs is crucial in the pathogenesis of SLE	(28)
	Juvenile dermatomyositis	Biomarker for tracking disease activity and treatment response prediction in juvenile dermatomyositis.	(75)
	Systemic Sclerosis	Expression is upregulated by type I IFNs.	(76)
	Viral infection	Cooperates with DAP12 to activate SHP2, recruits TRIM27 (an E3 ubiquitin ligase), and promotes TBK1 degradation via K48-linked ubiquitination to inhibit viral infection.	(77)
Siglec-2	Alzheimer’s disease and Neurodegenerativediseases	Neurons highly sialylated activating Siglec receptors signal via ITIM domains. Activates SHP-1/SHP-2 protein, thus inhibit microglial phagocytosis.	(78)
	Autoimmune disease.	CD22 regulates B cell activation and tolerance. Disruptions in these mechanisms can contribute to autoimmune diseases.	(79)
Siglec-3	Alzheimer’s disease	Inhibits amyloid β42 uptake by microglial cells.	(80)
	Neurodegenerative disease.	Neurons highly sialylated activating Siglec receptors signal via ITIM domains. Activates SHP-1/SHP-2 protein, thus inhibit microglial phagocytosis.	(78)
	Chronic Hepatitis B	Hepatitis virus (HBV) activated Siglec-3 on myeloid cells, leading to immunosuppression by ITIM phosphorylation and SHP-1/-2 recruitment via α2,6-biantennary sialoglycans on HBsAg.	(81)
Siglec-4 (MAG)	Cancer	Disrupts cell-cell and cell-to-ECM interactions. Immunosuppressive chemokines, immunological checkpoints, and tumor microenvironment (TME) signaling pathways involved.	(9)
Siglec-5	Pneumoniae	Pneumococcal NanA-mediated desialylation disrupts Siglec-5-TLR-2 interaction Reduces SHP-1 recruitment, leading to increased inflammation and cytotoxicity in infected macrophages.	(82)
Siglec-6	Cancer	Functions as a regulatory molecule that helps maintain immune homeostasis. Acts as a disease marker or therapeutic target in various pathologies, particularly blood cancers and allergic diseases.	(45)
Siglec-7	Acute myeloid leukemia	Serves as a glycolimmune checkpoint Interacts with a ganglioside (GD3) present on tumor cells. Impedes the capacity of natural killer cells to target and destroy the tumor.	(83)
Siglec-8	Allergic Asthma	Expression higher in asthma patients compared to healthy controls. Expressed on mast cells which are activated by crosslinking the FcϵRI receptor by IgEInduces rapid degranulation and releases of proteases, tumor necrosis factor (TNF), histamine, lipid mediators, cytokines, and chemokines.	(84)
Siglec-9	Glioblastoma Multiforme (GBM)	Expressed in several subpopulations of tumor-associated macrophages derived from monocytes. These macrophages are functionally adaptable and selectively accumulate in patients.	(85)
	Rheumatoid arthritis	Controls differentiation of Th17 and Treg cells. Soluble siglec-9 can suppress arthritis by inhibiting the NF- kB pathway, which leads to the suppression of M1 proinflammatory macrophages.	(37)
Siglec-10	Gastric cancer (GC)	Controls the immune-suppressing effect of tumor-associated macrophage regulator (TAM). Helps with the depletion of CD8+ T cells.	(10)
Siglec-11	Alzheimer’s disease	Highly sialylated neurons activate Siglec receptors by ITIM domains Activates SHP-1/SHP-2 protein, thus inhibiting microglial phagocytosis.	(86)
Siglec-12	Cardiovascular disorders	Plays a part in the negative control of macrophage signaling. SNP rs16982743 is linked to cardiovascular disorders based on treatment type (calcium channel blocker/beta-blocker).	(87)
Siglec-14	Chronic obstructive pulmonary disease (COPD)	Enhances inflammasome activation. Promotes the release of IL-1β by macrophages. Exhibits increase caspase-1 activation, IL-1β release, and pyroptosis after infection with group B Streptococcus (GBS).	(88)
Siglec-15	Osteoporosis, cancer, and infectious diseases	Binds to CD44 on osteoclast precursors. Activates a DAP12-SYK pathway that interacts with RANK-TRAF6, increasing downstream activation, including ERK and PI3K-AKT, and necessitating the presence of sialic acids.	(89)

## 5 Siglec protein interaction partners and associated diseases

Siglecs interact with multiple protein partners to regulate immune signaling, self-tolerance, and disease progression. These interactions influence endocytosis, cytokine production, antigen receptor signaling, and immune suppression Siglec-1/CD169 expressed mainly on macrophages, mediates sialic acid dependent interactions with diverse immune cells and participates in clathrin-mediated endocytosis (90). It preferentially recognizes α2,3-linked sialic acids and interacts with ligands such as CD43 on T cells, SR-BI, VAP-1, and tumor-associated sialyl-Tn antigens (91). Through a DAP12-SHP2-TRIM27-TBK1 axis, Siglec-1/CD169 suppresses type I interferon production, highlighting its dual role in immune regulation and pathology (77). Siglec-2 functions as a key inhibitory coreceptor of the B-cell receptor (BCR). Upon phosphorylation by Lyn kinase, Siglec-2 recruits SHP-1 phosphatase, leading to attenuation of BCR signaling and maintenance of B-cell tolerance. Dysregulation of this pathway contributes to autoimmune disease and B-cell malignancies (92).

In the central nervous system, Siglec-3 and TREM2 cooperatively regulate microglial activation and neuroinflammation. Their interaction modulates IL-1β related signaling pathways and genes involved in chemokine receptor function, implicating them as therapeutic targets in Alzheimer’s disease (93). Similarly, Siglec-4 interacts with NgR1 to inhibit neurite outgrowth, influencing neuronal regeneration and neuropsychiatric disorders (86). Siglec-5 and Siglec-14 recognize pathogen-associated sialic acids, particularly during bacterial infections, and signal through SHP-1/2 or DAP12-dependent pathways (94). Siglec-7 and Siglec-9 bind α2,8-linked sialic acids on immune and target cells, modulating NK-cell cytotoxicity (95). Siglec-10 interacts with the sialylated glycoprotein CD24 to suppress inflammatory responses, especially at immune-privileged sites such as the maternal fetal interface (33). Siglec-15 binds tumor-associated sialylated glycans, including sialyl-Tn, and signals through DAP12-SYK to induce TGF-β secretion, promoting an immunosuppressive tumor microenvironment. Collectively, Siglec protein interactions shape immune homeostasis and contribute to autoimmune, neurodegenerative, infectious, and malignant diseases.

## 6 Regulators of Siglec gene expression and function

Siglec expression and function are tightly regulated by transcription factors, microRNAs (miRNAs), epigenetic mechanisms, and environmental cues. Viral infection induced activation of the IFN/JAK/STAT1 pathway suppresses miR-27a, resulting in increased Siglec-1/CD169 expression and enhanced viral replication (77). STAT1 signaling also regulates Siglec-2 expression during B-cell differentiation in chronic lymphocytic leukemia (96). Hypoxia/ischemia drives microglial M1 polarization and upregulates CD33 and SHP-1, inhibiting TREM2-mediated phagocytosis and reducing amyloid clearance, leading to plaque accumulation. It also increases sialylated gangliosides (GM1, GM2, GM3, GD1), which act as ligands for inhibitory receptors such as Siglec-3 (97). In myeloma cells, IL-6 suppresses Siglec-3 expression by MYC-mediated repression of CEBPA (98). Several miRNAs directly target Siglecs, including miR-215 targeting Siglec-8 in Hirschsprung’s disease and miRNAs regulating Siglec-11 and Siglec-15 in glioblastoma (99). Regulatory elements within untranslated regions further modulate Siglec expression; the 3′-UTR of Siglec-15 promotes mRNA degradation, while the 5′-UTR inhibits translation. Epigenetic modifications, including histone changes and CpG methylation, also control Siglec-7 expression in NK cells. These findings highlight the complex regulatory networks governing Siglec function across diseases (100, 101).

## 7 Siglec mediated pathways in human diseases

Siglecs influence multiple signaling pathways involved in immune regulation, inflammation, neurodegeneration, autoimmunity, and cancer. Siglec-1/CD169 is strongly linked to type I interferon signaling and serves as a biomarker in systemic sclerosis, juvenile dermatomyositis, and other interferon-driven autoimmune diseases (12). Siglec-2 modulates BCR signaling to maintain immune tolerance, and its dysregulation contributes to autoimmune diseases such as systemic lupus erythematosus. In neurodegeneration, Siglec-3 suppresses microglial phagocytosis of amyloid-β, counterbalanced by TREM2-mediated activation, illustrating a finely tuned regulatory system in Alzheimer’s disease (92). Siglec-4 participates in MAPK and AKT signaling pathways essential for myelin integrity, with dysfunction linked to neuropathies. Siglec-15 signals through the DAP12-SYK/MAPK pathway, influencing osteoclastogenesis and immune suppression within the tumor microenvironment (14). Overall, Siglec mediated signaling pathways represent critical nodes in disease pathogenesis and offer promising opportunities for therapeutic intervention.

## 8 Challenges and future directions in Siglec-targeted therapies

Targeting the sialoglycan-Siglec axis has emerged as a promising cancer immunotherapy strategy. Siglecs serve as tumor antigens, with antibody drug conjugates targeting Siglec-2 (CD22) showing clinical efficacy, and CAR-T approaches co-targeting CD22/CD19. Siglec-3 is also being explored in acute myeloid leukemia (40, 102, 103). Siglecs additionally function as immune checkpoints. Blocking antibodies, such as the Siglec-15 inhibitor NC318, have demonstrated early clinical activity (104, 105), while inhibition of Siglec-7, -9, and -10 enhances NK cell function and macrophage-mediated phagocytosis (106, 107). Targeting sialoglycans represents an alternative approach, including glycoform-specific antibodies, inhibition of sialic acid biosynthesis, and enzymatic desialylation. Together, these strategies underscore the therapeutic potential of modulating the Siglec-glycan axis in cancer. Additional clinical and preclinical evidence on Siglec targeting is summarized in **Table 2**.

**Table 2:** Clinical and preclinical evidence targeting Siglecs gene.

Target Siglecs	Therapeutic agent	Strategy type	Disease indication	Stage	Key findings
Siglec-2 (CD22)	Inotuzumab ozogamicin	ADC	Acute lymphoblastic leukemia (ALL)	Approved/Clinical	Improved response rates in relapsed ALL (40).
Siglec-2 (CD22)	CD22/CD19 CAR-T cells	CAR-T therapy	B-cell malignancies	Clinical	Enhanced targeting and reduced relapse (103).
Siglec-3 (CD33)	Gemtuzumab ozogamicin	ADC	Acute myeloid leukemia (AML)	Approved/Clinical	Induces remission in AML patients (102).
Siglec-15	NC318	Monoclonal antibody (checkpoint inhibitor)	Solid tumors (e.g., NSCLC)	Clinical trials	Promotes anti-tumor immunity; early efficacy signals (104, 105).
Siglec-7	Blocking antibodies	Immune checkpoint blockade	Cancer	Preclinical	Enhances NK cell cytotoxicity (106).
Siglec-9	Blocking antibodies	Immune checkpoint blockade	Cancer	Preclinical	Improves T cell and NK cell responses (106).
Siglec-10	Anti-CD24/Siglec-10 axis inhibitors	Phagocytosis checkpoint targeting	Solid tumors	Preclinical	Enhances macrophage-mediated phagocytosis (107).
Multiple Siglecs	Sialic acid mimetics	Glycoengineering	Cancer	Preclinical	Reduces hypersialylation, boosts immunity (108).
Multiple Siglecs	Sialidase-conjugated antibodies	Enzymatic glycoengineering	Cancer	Preclinical/Early clinical	Enhances immune cell activation and tumor killing (109).

## 9 Conclusion

Through their interactions with many proteins and signaling pathways, siglecs, which are single-pass transmembrane proteins, are involved in various human diseases. The regulation of their expression and function is controlled by miRNAs and transcription factors, which have an impact on the initiation and advancement of many diseases. This review intends to offer a thorough summary of the role of Siglec family genes in human disorders by examining the scattered literature. This review also provides critical perspectives on the involvement of Siglecs in the progression and recuperation of diseases to clarify the molecular processes that relate to these interactions, with the goal of improving the knowledge of disease pathogenicity and assisting in the discovery of new Siglec targeting biomarkers and therapeutic approaches for the treatment of various disorders.

## References

[1] Läubli H Varki A . Sialic acid-binding immunoglobulin-like lectins (Siglecs) detect self-associated molecular patterns to regulate immune responses. Cell Mol Life sciences:CMLS. (2020) 77:593–605. doi: 10.1007/s00018-019-03288-x, PMID: 31485715 PMC7942692

[2] Zhao M Zhu Y Wang H Zhang W Mu W . Recent advances on N-acetylneuraminic acid: Physiological roles, applications, and biosynthesis. Synthetic Syst Biotechnol. (2023) 8:509–19. doi: 10.1016/j.synbio.2023.06.009, PMID: 37502821 PMC10369400

[3] Pillai S Netravali IA Cariappa A Mattoo H . Siglecs and immune regulation. Annu Rev Immunol. (2012) 30:357–92. doi: 10.1146/annurev-immunol-020711-075018, PMID: 22224769 PMC3781015

[4] Lin C-H Yeh Y-C Yang KD . Functions and therapeutic targets of Siglec-mediated infections, inflammations and cancers. J Formosan Med Association 120(1. (2021) Pt. 1):5–24. doi: 10.1016/j.jfma.2019.10.019, PMID: 31882261

[5] van Houtum EJH Büll C Cornelissen LAM Adema GJ . Siglec signaling in the tumor microenvironment. Front Immunol. (2021) 12:790317. doi: 10.3389/fimmu.2021.790317, PMID: 34966391 PMC8710542

[6] Schwarz F Landig CS Siddiqui S Secundino I Olson J Varki N . Paired Siglec receptors generate opposite inflammatory responses to a human-specific pathogen. EMBO J. (2017) 36:751–60. doi: 10.15252/embj.201695581, PMID: 28100677 PMC5350563

[7] Feng H Feng J Han X Ying Y Lou W Liu L . The potential of Siglecs and sialic acids as biomarkers and therapeutic targets in tumor immunotherapy. Cancers. (2024) 16:289. 38254780 10.3390/cancers16020289 PMC10813689

[8] Saini P Adeniji OS Abdel-Mohsen M . Inhibitory Siglec–sialic acid interactions in balancing immunological activation and tolerance during viral infections. eBioMedicine. (2022) 86:104354. doi: 10.1016/j.ebiom.2022.104354, PMID: 36371982 PMC9663867

[9] Lim J Sari-Ak D Bagga T . Siglecs as Therapeutic Targets in Cancer. Biology. (2021) 10:1178. doi: 10.3390/biology10111178, PMID: 34827170 PMC8615218

[10] Wang X Liu D Ning Y Liu J Wang X Tu R . Siglec-9 is upregulated in rheumatoid arthritis and suppresses collagen-induced arthritis through reciprocal regulation of Th17-/Treg-cell differentiation. Scandinavian J Immunol. (2017) 85:433–40. doi: 10.1111/sji.12543, PMID: 28273363

[11] Ostendorf L Dittert P Biesen R Duchow A Stiglbauer V Ruprecht K . SIGLEC1 (CD169): A marker of active neuroinflammation in the brain but not in the blood of multiple sclerosis patients. Sci Rep. (2021) 11:10299. 33986412 10.1038/s41598-021-89786-0 PMC8119413

[12] Linnartz-Gerlach B Mathews M Neumann H . Sensing the neuronal glycocalyx by glial sialic acid–binding immunoglobulin-like lectins. Neuroscience. (2014) 275:113–24. doi: 10.1016/j.neuroscience.2014.05.061, PMID: 24924144

[13] Angata T Varki A . Discovery, classification, evolution and diversity of Siglecs. Mol aspects Med. (2023) 90:101117. doi: 10.1016/j.mam.2022.101117, PMID: 35989204 PMC9905256

[14] Angata T Ishii T Motegi T Oka R Taylor RE Soto PC . Loss of Siglec-14 reduces the risk of chronic obstructive pulmonary disease exacerbation. Cell Mol Life sciences: CMLS. (2013) 70:3199–210. doi: 10.1007/s00018-013-1311-7, PMID: 23519826 PMC3718857

[15] Fan Y Sun L He J Chen Y Ma H Ding H . Siglec15 in blood system diseases: from bench to bedside. Front Immunol. (2024) 15:1490505. doi: 10.3389/fimmu.2024.1490505, PMID: 39697338 PMC11652361

[16] Cozac-Szőke AR Tinca AC Negovan A Vilaia A Cozac DA Cocuz IG . Comprehensive Analysis of SIGLEC-15 and PD-L1 Expression Identifies Distinct Prognostic Profiles in Gastric Cancer. Int J Mol Sci. (2025) 26:8637. doi: 10.3390/ijms26178637, PMID: 40943560 PMC12429166

[17] Thiesler H Hildebrandt H . Polysialic acid–Siglec immune checkpoints of microglia and macrophages: Perspectives for therapeutic intervention. Neural Regeneration Res. (2026) 21:661–2. doi: 10.4103/NRR.NRR-D-24-01195, PMID: 39688555 PMC12220714

[18] Mantuano NR Läubli H . Sialic acid and Siglec receptors in tumor immunity and immunotherapy. Semin Immunol. (2024) 74-75:101893. doi: 10.1016/j.smim.2024.101893, PMID: 39427573

[19] Coccimiglio M Chiodo F van Kooyk Y . The sialic acid–Siglec immune checkpoint: An opportunity to enhance immune responses and therapy effectiveness in melanoma. Br J Dermatol. (2024) 190:627–35. doi: 10.1093/bjd/ljad517, PMID: 38197441

[20] Boelaars K van Kooyk Y . Targeting myeloid cells for cancer immunotherapy: Siglec-7/9/10/15 and their ligands. Trends Cancer. (2024) 10:230–41. doi: 10.1016/j.trecan.2023.11.009, PMID: 38160071

[21] Sun J Lu Q Sanmamed MF Wang J . Siglec-15 as an Emerging Target for Next-generation Cancer Immunotherapy. Clin Cancer Res an Off J Am Assoc Cancer Res. (2021) 27:680–8. doi: 10.1158/1078-0432.CCR-19-2925, PMID: 32958700 PMC9942711

[22] Wieboldt R Sandholzer M Carlini E Lin CW Börsch A Zingg A . Engagement of sialylated glycans with Siglec receptors on suppressive myeloid cells inhibits anticancer immunity via CCL2. Cell Mol Immunol. (2024) 21:495–509. doi: 10.1038/s41423-024-01142-0, PMID: 38448555 PMC11061307

[23] Gianchecchi E Arena A Fierabracci A . Sialic Acid-Siglec Axis in Human Immune Regulation, Involvement in Autoimmunity and Cancer and Potential Therapeutic Treatments. Int J Mol Sci. (2021) 22:5774. doi: 10.3390/ijms22115774, PMID: 34071314 PMC8198044

[24] Macauley MS Crocker PR Paulson JC . Siglec-mediated regulation of immune cell function in disease. Nat Rev Immunol. (2014) 14:653–66. doi: 10.1038/nri3737, PMID: 25234143 PMC4191907

[25] Chang YC Nizet V . The interplay between Siglecs and sialylated pathogens. Glycobiology. (2014) 24:818–25. doi: 10.1093/glycob/cwu067, PMID: 24996821 PMC4168292

[26] Bandala-Sanchez E Bediaga NG Naselli G Neale AM Harrison LC . Siglec-10 expression is up-regulated in activated human CD4+ T cells. Hum Immunol. (2020) 81:101–4. doi: 10.1016/j.humimm.2020.01.009, PMID: 32046870

[27] Kukan EN Fabiano GL Cobb BA . Siglecs as modulators of macrophage phenotype and function. Semin Immunol. (2024) 73:101887. doi: 10.1016/j.smim.2024.101887, PMID: 39357273

[28] Mirioğlu Ş. Çınar S Uludağ Ö. Gürel E Varelci S Özlük Y . Serum and urine interferon-inducible protein 10, galectin-9, and SIGLEC-1 as biomarkers of disease activity in systemic lupus erythematosus. Turkish J Med Sci. (2024) 54:391–400. doi: 10.55730/1300-0144.5804, PMID: 39050398 PMC11265893

[29] Ritter J Szelinski F Aue A Stefanski A-L Rincon-Arevalo H Chen Y . Elevated unphosphorylated STAT1 and IRF9 in T and B cells of primary Sjögren’s syndrome: Novel biomarkers for disease activity and subsets. J Autoimmun. (2024) 147:103243. doi: 10.1016/j.jaut.2024.103243, PMID: 38788537

[30] Guo M Guo H Zhu J Wang F Chen J Wan C . A novel subpopulation of monocytes with a strong interferon signature indicated by SIGLEC-1 is present in patients with recent-onset type 1 diabetes. Diabetologia. (2024) 67:623–40. doi: 10.1007/s00125-024-06098-4, PMID: 38349399

[31] Xiang W Wang K Han L Wang Z Zhou Z Bai S . CD22 blockade aggravates EAE and its role in microglia polarization. CNS Neurosci Ther. (2024) 30:e14736. doi: 10.1111/cns.14736, PMID: 38739106 PMC11090149

[32] Okuzono Y Miyakawa S Itou T Sagara M Iwata M Ishizuchi K . B-cell immune dysregulation with low soluble CD22 levels in refractory seronegative myasthenia gravis. Front Immunol. (2024) 15:1382320. doi: 10.3389/fimmu.2024.1382320, PMID: 38711503 PMC11071663

[33] Liu Y Li H Yu H Wang F Cao H Jia J . Deciphering prognostic value of CD22 and its contribution to suppression of proinflammatory cytokines production in patients with IgA nephropathy. Immunol Lett. (2023) 255:40–6. doi: 10.1016/j.imlet.2023.02.007, PMID: 36848961

[34] Huang Y-J Lee J-J Fan W-L Hsu C-W Tsai N-W Lu C-H . A CD33 frameshift variant is associated with neuromyelitis optica spectrum disorders. Biomed Journal 44(6. (2021) Suppl. 1):S93–S100. doi: 10.1016/j.bj.2020.07.007, PMID: 35735085 PMC9038945

[35] Flores R Zhang P Wu W Wang X Ye P Zheng P . Siglec genes confer resistance to systemic lupus erythematosus in humans and mice. Cell Mol Immunol. (2019) 16:154–64. doi: 10.1038/cmi.2017.160, PMID: 29503442 PMC6355849

[36] Brzezicka KA Paulson JC . Impact of Siglecs on autoimmune diseases. Mol aspects Med. (2023) 90:101140. doi: 10.1016/j.mam.2022.101140, PMID: 36055802 PMC9905255

[37] Wang X Liu M Zhang J Brown NK Zhang P Zhang Y . CD24-Siglec axis is an innate immune checkpoint against metaflammation and metabolic disorder. Cell Metab. (2022) 34:1088–1103.e6. doi: 10.1016/j.cmet.2022.07.005, PMID: 35921817 PMC9393047

[38] Takamiya R Ohtsubo K Takamatsu S Taniguchi N Angata T . The interaction between Siglec-15 and tumor-associated sialyl-Tn antigen enhances TGF-β secretion from monocytes/macrophages through the DAP12-Syk pathway. Glycobiology. (2013) 23:178–87. doi: 10.1093/glycob/cws139, PMID: 23035012

[39] Mauvais F-X van Endert P . Siglec-1⁺ macrophages in anti-tumor immunity. Immunol Lett. (2026) 277:107108. doi: 10.1016/j.imlet.2025.107108, PMID: 41205710

[40] Rubinstein JD O'Brien MM . Inotuzumab ozogamicin in B-cell precursor acute lymphoblastic leukemia: efficacy, toxicity, and practical considerations. Front Immunol. (2023) 14:1237738. doi: 10.3389/fimmu.2023.1237738, PMID: 37600823 PMC10435844

[41] Chen J Pan Z Han L Liu J Yue Y Xiao X . Binding domain on CD22 molecules contributing to the biological activity of T cell-engaging bispecific antibodies. Heliyon. (2023) 9:e17960. doi: 10.1016/j.heliyon.2023.e17960, PMID: 37456045 PMC10344817

[42] Silva HJ Martin G Birocchi F Wehrli M Kann MC Supper V . CD70 CAR T cells secreting an anti-CD33/anti-CD3 dual-targeting antibody overcome antigen heterogeneity in AML. Blood. (2025) 145:720–31. doi: 10.1182/blood.2023023210, PMID: 39571145 PMC11863708

[43] Li YR Fang Y Niu S Zhu Y Chen Y Lyu Z . Allogeneic CD33-directed CAR-NKT cells for the treatment of bone marrow-resident myeloid malignancies. Nat Commun. (2025) 16:1248. doi: 10.1038/s41467-025-56270-6, PMID: 39893165 PMC11787387

[44] Warner van Dijk FA Tong O O'Neil TR Bertram KM Hu K Baharlou H . Characterising plasmacytoid and myeloid AXL+ SIGLEC-6+ dendritic cell functions and their interactions with HIV. PloS Pathog. (2024) 20:e1012351. doi: 10.1371/journal.ppat.1012351, PMID: 38924030 PMC11233022

[45] Nunes J Tafesse R Mao C Purcell M Mo X Zhang L . Siglec-6 as a therapeutic target for cell migration and adhesion in chronic lymphocytic leukemia. Nat Commun. (2024) 15:5180. doi: 10.1038/s41467-024-48678-3, PMID: 38890323 PMC11189495

[46] Galaski J Rishiq A Liu M Bsoul R Bergson A Lux R . Fusobacterium nucleatum subsp. nucleatum RadD binds Siglec-7 and inhibits NK cell–mediated cancer cell killing. iScience. (2024) 27:110157. doi: 10.1016/j.isci.2024.110157, PMID: 38952680 PMC11215305

[47] Eisenberg V Hoogi S Katzman E Ben Haim N Zur-Toledano R Radman M . Targeting Tumor-Associated Sialic Acids Using Chimeric Switch Receptors Based on Siglec-9 Enhances the Antitumor Efficacy of Engineered T Cells. Cancer Immunol Res. (2024) 12:1380–91. doi: 10.1158/2326-6066.CIR-23-0823, PMID: 39037052

[48] Yang P Jiang Y Chen R Yang J Liu M Huang X . Prognostic and immune infiltration implications of SIGLEC9 in SKCM. Diagn Pathol. (2024) 19:112. doi: 10.1186/s13000-024-01536-8, PMID: 39153970 PMC11330613

[49] Medina E Mason C Tran TH Juliá EP Ming Q Taibi LM . Structural basis for sialoglycan recognition by the immune inhibitory receptor Siglec-10. Structure (London Engl. (2026) 1993):S0969–2126(26)00032-8. doi: 10.1016/j.str.2026.01.018, PMID: 41747717

[50] Daly J Piatnitca L Al-Seragi M Krishnamoorthy V Wisnovsky S . CRISPR activation screens map the genomic landscape of cancer glycome remodeling. Cell Genomics. (2026) 6:101139. doi: 10.1016/j.xgen.2026.101139, PMID: 41610854 PMC13069869

[51] Saini P Mirji G Islam SMS Simons LM Bhat SA Bonfanti AP . Targeting Interactions Between Siglec-10 and α3β1 Integrin Enhances Macrophage-Mediated Phagocytosis of Pancreatic Cancer. Cancer Res. (2026) 86:99–115. doi: 10.1158/0008-5472.CAN-25-0977, PMID: 41182080 PMC12671567

[52] Huang S Zhang X Wei Y Xiao Y . Checkpoint CD24 function on tumor and immunotherapy. Front Immunol. (2024) 15:1367959. doi: 10.3389/fimmu.2024.1367959, PMID: 38487533 PMC10937401

[53] Fang J Lin L Cao Y Tan J Liang Y Xiao X . Targeting the CD24–Siglec-10 axis: A potential strategy for cancer immunotherapy. Bio Integration. (2024) 5:1–14. doi: 10.15212/bioi-2023-0022

[54] Cuello HA Sinha S Verhagen AL Varki N Varki A Ghosh P . Human-specific elimination of epithelial Siglec-XII suppresses the risk of inflammation-driven colorectal cancers. JCI Insight. (2024), e181539. doi: 10.1172/jci.insight.181539, PMID: 38990656 PMC11343606

[55] Zhang X Shi J Jin S Wang R Li M Zhang Z . Metabolic landscape of head and neck squamous cell carcinoma informs a novel kynurenine/Siglec-15 axis in immune escape. Cancer Commun. (2024) 44:670–94. doi: 10.1002/cac2.12545, PMID: 38734931 PMC11194450

[56] Sun H Du Q Xu Y Rao C Xu L Yang J . The expression characteristic and prognostic role of Siglec-15 in lung adenocarcinoma. Clin Respir J. (2024) 18:e13772. doi: 10.1111/crj.13772, PMID: 38725348 PMC11082535

[57] Wißfeld J Abou Assale T Cuevas-Rios G Liao H Neumann H . Therapeutic potential to target sialylation and SIGLECs in neurodegenerative and psychiatric diseases. Front Neurol. (2024) 15:1330874. doi: 10.3389/fneur.2024.1330874, PMID: 38529039 PMC10961342

[58] Bu X-L Sun P-Y Fan D-Y Wang J Sun H-L Cheng Y . Science Advances, 8 (2022). doi: 10.1126/sciadv.abm5667, PMID: PMC10938586 35363517

[59] Pluvinage JV Sun J Claes C Flynn RA Haney MS Iram T . The CD22–IGF2R interaction is a therapeutic target for microglial lysosome dysfunction in Niemann–Pick type C. Sci Trans Med. (2021) 13:eabg2919. doi: 10.1126/scitranslmed.abg2919, PMID: 34851695 PMC9067636

[60] Vu HN Situ AJ Dai X Ulmer TS . Structure of the CD33 Receptor and Implications for the Siglec Family. Biochemistry. (2025) 64:1450–62. doi: 10.1021/acs.biochem.4c00864, PMID: 40067740 PMC12002911

[61] Gonzalez-Gil A Porell RN Fernandes SM Maenpaa E Li TA Li T . Human brain sialoglycan ligand for CD33, a microglial inhibitory Siglec implicated in Alzheimer's disease. J Biol Chem. (2022) 298:101960. doi: 10.1016/j.jbc.2022.101960, PMID: 35452678 PMC9130525

[62] Herzog S Fragkou PC Arneth BM Mkhlof S Skevaki C . Myeloid CD169/Siglec1: An immunoregulatory biomarker in viral disease. Front Med. (2022) 9:979373. doi: 10.3389/fmed.2022.979373, PMID: 36213653 PMC9540380

[63] Tsai TY Huang MT Sung PS Peng CY Tao MH Yang HI . SIGLEC-3 (CD33) serves as an immune checkpoint receptor for HBV infection. J Clin Invest. (2021) 131:e141965. doi: 10.1172/JCI141965, PMID: 34060491 PMC8159688

[64] Cardenas AJ Thomas KS Broden MW Ferraro NJ Pires MM John CM . Neisseria gonorrhoeae scavenges host sialic acid for Siglec-mediated, complement-independent suppression of neutrophil activation. mBio. (2024) 15:e00119–24. doi: 10.1128/mbio.00119-24, PMID: 38587424 PMC11078009

[65] Doehn J-M Tabeling C Biesen R Saccomanno J Madlung E Pappe E . CD169/SIGLEC1 is expressed on circulating monocytes in COVID-19 and expression levels are associated with disease severity. Infection. (2021) 49:757–62. doi: 10.1007/s15010-021-01606-9, PMID: 33825125 PMC8023546

[66] Jalloh S Olejnik J Berrigan J Nisa A Suder EL Akiyama H . CD169-mediated restrictive SARS-CoV-2 infection of macrophages induces pro-inflammatory responses. PloS Pathog. (2022) 18:e1010479. doi: 10.1371/journal.ppat.1010479, PMID: 36279285 PMC9632919

[67] Martínez-Diz S Marín-Benesiu F . Relevance of TMPRSS2, CD163/CD206, and CD33 in clinical severity stratification of COVID-19. Front Immunol. (2023) 13:1094644. doi: 10.3389/fimmu.2022.1094644, PMID: 36969980 PMC10031647

[68] Chang LY Liang SY Lu SC Tseng HC Tsai HY Tang CJ . Molecular Basis and Role of Siglec-7 Ligand Expression on Chronic Lymphocytic Leukemia B Cells. Front Immunol. (2022) 13:840388. doi: 10.3389/fimmu.2022.840388, PMID: 35711441 PMC9195294

[69] Gebremeskel S Schanin J . Mast Cell and Eosinophil Activation Are Associated With COVID-19 and TLR-Mediated Viral Inflammation: Implications for an Anti-Siglec-8 Antibody. Front Immunol. (2021) 12:650331. doi: 10.3389/fimmu.2021.650331, PMID: 33777047 PMC7988091

[70] Saini P Adeniji OS Bordoloi D Kinslow J Martinson J Parent DM . Siglec-9 Restrains Antibody-Dependent Natural Killer Cell Cytotoxicity against SARS-CoV-2. mBio. (2023) 14:e0339322. doi: 10.1128/mbio.03393-22, PMID: 36728420 PMC9973332

[71] Delaveris CS Wilk AJ Riley NM Stark JC Yang SS Rogers AJ . Synthetic Siglec-9 agonists inhibit neutrophil activation associated with COVID-19. ACS Cent Sci. (2021) 7:650–7. doi: 10.1021/acscentsci.0c01669, PMID: 34056095 PMC8009098

[72] He X Zhang X Wu B . The receptor binding domain of SARS-CoV-2 Omicron subvariants targets Siglec-9 to decrease its immunogenicity by preventing macrophage phagocytosis. Nat Immunol. (2024) 25:622–32. doi: 10.1038/s41590-024-01776-2, PMID: 38454157

[73] Boz V Tesser A Burlo F Donadel N Pastore S Amaddeo A . Siglec-1, an easy and contributory inflammation marker in rheumatology. Clin Trans Immunol. (2024) 13:e1520. doi: 10.1002/cti2.1520, PMID: 38939726 PMC11208081

[74] Xiong Y-S Cheng Y Lin Q-S Wu A-L Yu J Li C . Increased expression of Siglec-1 on peripheral blood monocytes and its role in mononuclear cell reactivity to autoantigen in rheumatoid arthritis. Rheumatology. (2014) 53:250–9. doi: 10.1093/rheumatology/ket342, PMID: 24196391

[75] Lerkvaleekul B Veldkamp SR van der Wal MM Schatorjé EJH Kamphuis SSM Van den Berg JM . Siglec-1 expression on monocytes is associated with the interferon signature in juvenile dermatomyositis and can predict treatment response. Rheumatology. (2022) 61:2144–55. doi: 10.1093/rheumatology/keab601, PMID: 34387304 PMC9071568

[76] Höppner J Casteleyn V Biesen R Rose T Windisch W Burmester GR . SIGLEC-1 in systemic sclerosis: A useful biomarker for differential diagnosis. Pharmaceuticals. (2022) 15:1198. 36297311 10.3390/ph15101198 PMC9610402

[77] Zheng Q Hou J Zhou Y Yang Y Xie B Cao X . Siglec1 suppresses antiviral innate immune response by inducing TBK1 degradation via the ubiquitin ligase TRIM27. Cell Res. (2015) 25:1121–36. doi: 10.1038/cr.2015.108, PMID: 26358190 PMC4650625

[78] Butler CA Popescu AS Kitchener EJA Allendorf DH Puigdellívol M Brown GC . Microglial phagocytosis of neurons in neurodegeneration, and its regulation. J Neurochemistry. (2021) 158:621–39. doi: 10.1111/jnc.15327, PMID: 33608912

[79] Clark EA Giltiay NV . CD22: A regulator of innate and adaptive B cell responses and autoimmunity. Front Immunol. (2018) 9:2235. doi: 10.3389/fimmu.2018.02235, PMID: 30323814 PMC6173129

[80] Javor J Ďurmanová V Párnická Z . Association of CD33 rs3865444:C˃A polymorphism with a reduced risk of late-onset Alzheimer's disease in Slovaks is limited to subjects carrying the APOE ϵ4 allele. Int J Immunogenet. (2020) 47:397–405. doi: 10.1111/iji.12489, PMID: 32333488

[81] Tsung-Yu T Huang . SIGLEC-3 (CD33) serves as an immune checkpoint receptor for HBV infection. J Clin Invest. (2021) 131. doi: 10.1172/JCI141965, PMID: 34060491 PMC8159688

[82] Tseng Y-W Chang C-C Chang Y-C . Novel virulence role of pneumococcal NanA in host inflammation and cell death through the activation of inflammasome and the caspase pathway. Front Cell Infection Microbiol. (2021) 11:613195. doi: 10.3389/fcimb.2021.613195, PMID: 33777832 PMC7991587

[83] Haas Q Markov N . Siglec-7 represents a glyco-immune checkpoint for non-exhausted effector memory CD8+ T cells with high functional and metabolic capacities. Front Immunol. (2022) 13:996746. doi: 10.3389/fimmu.2022.996746, PMID: 36211376 PMC9540514

[84] Youngblood BA Leung J Falahati R Williams J Schanin J Brock EC . Discovery, Function, and Therapeutic Targeting of Siglec-8. Cells. (2021) 10:19. doi: 10.3390/cells10010019, PMID: 33374255 PMC7823959

[85] Mei Y Wang X Zhang J Liu D He J Huang C . Siglec-9 acts as an immune-checkpoint molecule on macrophages in glioblastoma, restricting T-cell priming and immunotherapy response. Nat Cancer. (2023) 4:1273–91. doi: 10.1038/s43018-023-00598-9, PMID: 37460871

[86] Siddiqui SS Matar R Merheb M Hodeify R Vazhappilly CG Marton J . Siglecs in Brain Function and Neurological Disorders. Cells. (2019) 8:1125. doi: 10.3390/cells8101125, PMID: 31546700 PMC6829431

[87] Angata T . Associations of genetic polymorphisms of Siglecs with human diseases. Glycobiology. (2014) 24:785–93. doi: 10.1093/glycob/cwu043, PMID: 24841380

[88] Tsai C-M Riestra AM Ali SR Fong JJ Liu JZ Hughes G . Siglec-14 enhances NLRP3-inflammasome activation in macrophages. J Innate Immun. (2020) 12:333–43. doi: 10.1159/000504323, PMID: 31805552 PMC7383293

[89] Angata T . Siglec-15: a potential regulator of osteoporosis, cancer, and infectious diseases. J Biomed Sci. (2020) 27:10. doi: 10.1186/s12929-019-0610-1, PMID: 31900164 PMC6941304

[90] Ronco I Horvat B . Role of myeloid cells expressing CD169/Siglec-1 in host-pathogen interactions. FEMS Microbiol Rev. (2026) 50:fuag013. doi: 10.1093/femsre/fuag013, PMID: 41854333 PMC13070564

[91] Kawasaki N Vela JL Nycholat CM Rademacher C Khurana A Van Rooijen N . Targeted delivery of lipid antigen to macrophages via the CD169/sialoadhesin endocytic pathway induces robust invariant natural killer T cell activation. Proc Natl Acad Sci United States America. (2013) 110:7826–31. doi: 10.1073/pnas.1219888110, PMID: 23610394 PMC3651435

[92] Nitschke L . CD22 and Siglec-G regulate inhibition of B-cell signaling by sialic acid ligand binding and control B-cell tolerance. Glycobiology. (2014) 24:807–17. doi: 10.1093/glycob/cwu066, PMID: 25002414

[93] Salminen A Kaarniranta K Kauppinen A . Hypoxia/ischemia impairs CD33 (Siglec-3)/TREM2 signaling: Potential role in Alzheimer’s pathogenesis. Neurochemistry Int. (2021) 150:105186. doi: 10.1016/j.neuint.2021.105186, PMID: 34530055

[94] Ali SR Fong JJ Carlin AF Busch TD Linden R Angata T . Siglec-5 and Siglec-14 are polymorphic paired receptors that modulate neutrophil and amnion signaling responses to group B Streptococcus. J Exp Med. (2014) 211:1231–42. doi: 10.1084/jem.20131853, PMID: 24799499 PMC4042635

[95] Jandus C Boligan KF Chijioke O Liu H Dahlhaus M Jalloh S . CD169-mediated restrictive SARS-CoV-2 infection of macrophages induces pro-inflammatory responses. PloS Pathog. (2022) 18:e1010479. doi: 10.1371/journal.ppat.1010479, PMID: 36279285 PMC9632919

[96] Battle TE Frank DA . STAT1 mediates differentiation of chronic lymphocytic leukemia cells in response to bryostatin 1. Blood. (2003) 102:3016–24. doi: 10.1182/blood-2002-09-2972, PMID: 12855573

[97] Mitroshina EV Vedunova MV . ‘The role of oxygen homeostasis and the HIF-1 factor in the development of neurodegeneration’. Int J Mol Sci. (2024) 25:4581. 38731800 10.3390/ijms25094581 PMC11083463

[98] Shamsasenjan K Otsuyama K Abroun S Iqbal MS Mahmoud MS Asaoku H . IL-6–induced activation of MYC is responsible for the down-regulation of CD33 expression in CD33⁺ myeloma cells. Int J Hematol. (2009) 89:310–8. doi: 10.1007/s12185-009-0256-y, PMID: 19259613

[99] Lei H Li H Xie H Du C Xia Y Tang W . Role of miR-215 in Hirschsprung’s disease pathogenesis by targeting SIGLEC-8. Cell Physiol Biochem. (2016) 40:1646–55. doi: 10.1159/000453214, PMID: 28006787

[100] Wang L Li R Lai X Zhang X Chen H Zhao W . Mapping regulatory elements within 5′ and 3′ UTRs of SIGLEC15 using a reporter system. Mol Biol. (2022) 56:406–16. doi: 10.1134/S0026893322030141 35621101

[101] Huang H Su S Chiou T Lin Y Shih Y Wu Y . DNA methylation–mediated Siglec-7 regulation in natural killer cells via two 5′ promoter CpG sites. Immunology. (2020) 160:38–51. doi: 10.1111/imm.13179, PMID: 32027025 PMC7160663

[102] Kantarjian HM Boissel N Papayannidis C Luskin MR Stelljes M Advani AS . Inotuzumab ozogamicin in adult acute lymphoblastic leukemia: Development, current status, and future directions. Cancer. (2024) 130:3631–46. doi: 10.1002/cncr.35505, PMID: 39093036 PMC12533514

[103] Angenendt L Mikesch J-H Schliemann C . Emerging antibody-based therapies for the treatment of acute myeloid leukemia. Cancer Treat Rev. (2022) 108:102409. doi: 10.1016/j.ctrv.2022.102409, PMID: 35605472

[104] Jiang KY Qi LL Liu XB Wang Y Wang L . Prognostic value of Siglec-15 expression in patients with solid tumors: A meta-analysis. Front Oncol. (2023) 12:1073932. doi: 10.3389/fonc.2022.1073932, PMID: 36713548 PMC9875589

[105] Ren X . Immunosuppressive checkpoint Siglec-15: a vital new piece of the cancer immunotherapy jigsaw puzzle. Cancer Biol Med. (2019) 16:205–10. doi: 10.20892/j.issn.2095-3941.2018.0141, PMID: 31516742 PMC6713637

[106] Purohit A Joshi I Bhojnagarwala PS Boyer JD Kim JJ Weiner DB . Siglecs in immunotherapy: Current clinical landscape and prospects. Trends Pharmacol Sci. (2025). doi: 10.1016/j.tips.2025.11.004, PMID: 41365751

[107] Li X Tian W Jiang Z Song Y Leng X Yu J . Targeting CD24/Siglec-10 signal pathway for cancer immunotherapy: recent advances and future directions. Cancer immunology immunotherapy: CII. (2024) 73:31. doi: 10.1007/s00262-023-03606-0, PMID: 38279998 PMC10821995

[108] Decloquement M van Houtum . Writers and readers of sialylation in immunoregulation in cancer. J Biol Chem. (2026) 302:111249. doi: 10.1016/j.jbc.2026.111249, PMID: 41651416 PMC12962172

[109] Li Y Chen H Gao J Wu P Hong S . Glycoengineering in antigen-specific immunotherapies. Curr Opin Chem Biol. (2024) 81:102503. doi: 10.1016/j.cbpa.2024.102503, PMID: 39053235 PMC11921784

